# Assessing corneal cross-linking with reverberant 3D optical coherence elastography

**DOI:** 10.1117/1.JBO.27.2.026003

**Published:** 2022-02-14

**Authors:** Gary R. Ge, Behrouz Tavakol, David B. Usher, Desmond C. Adler, Jannick P. Rolland, Kevin J. Parker

**Affiliations:** a University of Rochester, The Institute of Optics, Rochester, New York, United States; b Glaukos Corporation, San Clemente, California, United States; c University of Rochester, Department of Biomedical Engineering, Rochester, New York, United States; d University of Rochester, Center for Visual Science, Rochester, New York, United States; e University of Rochester, Department of Electrical and Computer Engineering, Rochester, New York, United States

**Keywords:** elastography, optical coherence tomography, cornea, cross-linking

## Abstract

**Significance:**

Corneal cross-linking (CXL) is a well-known procedure for treating certain eye disorders such as keratoconus. However, characterization of the biomechanical changes in the cornea as a result of this procedure is still under active research. Specifically, there is a clinical need for high-resolution characterization of individual corneal layers.

**Aim:**

A high-resolution elastography method in conjunction with a custom optical coherence tomography system is used to track these biomechanical changes in individual corneal layers. Pre- and post-treatment analysis for both low-dose and high-dose CXL experiments are performed.

**Approach:**

A recently developed elastography technique that utilizes the theory of reverberant shear wave fields, with optical coherence tomography as the modality, is applied to pig corneas *ex vivo* to evaluate elasticity changes associated with corneal CXL. Sets of low-dose and high-dose CXL treatments are evaluated before and after treatments with three pairs of pig corneas per experiment.

**Results:**

The reverberant three-dimensional (3D) optical coherence elastography (OCE) technique can identify increases in elasticity associated with both low-dose and high-dose CXL treatments. There is a notable graphical difference between low-dose and high-dose treatments. In addition, the technique is able to identify which layers of the cornea are potentially affected by the CXL procedure and provides insight into the nonlinearity of the elasticity changes.

**Conclusions:**

The reverberant 3D OCE technique can identify depth-resolved changes in elasticity of the cornea associated with CXL procedures. This method could be translated to assess and monitor CXL efficacy in various clinical settings.

## 1 Introduction

Keratoconus is a corneal disorder characterized by progressive thinning and a cone-like protrusion of the cornea.^1^ It is estimated to affect 50 to 230 per 100,000 individuals.^1^^–^^3^ One well-known treatment for keratoconus is corneal cross-linking (CXL).^4^^–^^6^ CXL utilizes riboflavin (vitamin B2) and ultraviolet-A (UV-A) light to strengthen collagen bonds in the cornea.^7^ Since the biomechanical properties of the cornea are significantly altered with this treatment, the elasticity of the cornea is a measurement of great interest. Numerous recent studies have been performed in assessing CXL using ultrasound elastography and optical coherence elastography (OCE).^8^^–^^17^ However, few have demonstrated high-resolution results that can characterize the CXL treatment in individual layers of the cornea.

In this study, we leverage a recently developed technique known as reverberant three-dimensional (3D) optical coherence elastography (Rev3D-OCE).^18^ This method was shown to map layers of the cornea with superior discrimination versus depth as compared with earlier Lamb wave propagation techniques. We apply Rev3D-OCE to assess the elasticity of the cornea pre- and post-CXL treatments in porcine corneas *ex vivo*. We find that CXL treatments increase the stiffness of the corneas, within a proximal zone centered around the surface treatment area. In addition, an increased elasticity can be profiled as a function of depth. The results reported demonstrate that Rev3D-OCE is a valid high-resolution method for detecting these elastic changes and would be able to better characterize various CXL procedures and protocols than conventional methods.

## 2 Methods

### 2.1 Sample Preparation

Three pairs of porcine eyes (six total) were procured (Pel-Freez, LLC, Rogers, Arkansas) for each set of experiments. All experiments were performed within 1 day of collection, and only intact eyes with non-damaged corneas were used. The eyes were allowed to warm to room temperature of 22°C in balanced salt solutions (BSS). Surrounding adipose and muscular tissues were removed before placing the eye in a custom-built holder. The epithelium of the corneas was gently removed using a scalpel right before application of the CXL drug. No scalding was performed. A needle was connected to an intravenous (IV) fluid bag containing BSS through an irrigation line. The needle was inserted through the holder into the eye to maintain an intraocular pressure of 15 mmHg. All eyes were irrigated with BSS at constant intervals to maintain hydration. All eyes were scanned using Rev3D-OCE before and after the CXL treatments.

For each eye, 2 to 3 drops of riboflavin solution (Vibex Rapid, Glaukos Corp.) were applied to the entire corneal surface in 20 s intervals for 10 min, with subsequent corneal rinsing with BSS. A custom illumination device applied a pulsed UV-A treatment (1 s period) with a treatment aperture of 3 mm and an irradiance of $30\;\mathrm{mW}/{\mathrm{cm}}^{2}$. Low-dose and high-dose CXL treatments were performed with doses of 5.4 and $\text{15 }J/{\mathrm{cm}}^{2}$, respectively. Treatment zones are aimed at the corneal center using a camera in the custom illumination device and span ∼3  mm in diameter. Negative control experiments were also performed whereby all the treatment protocol remained the same except the UV-A light was off during the treatment time. These sham experiments enabled us to assess the effect of crosslinking on the stiffness while mitigating the effect of drug application and dehydration during the treatment.

### 2.2 Experimental Setup

**Fig. 1:**
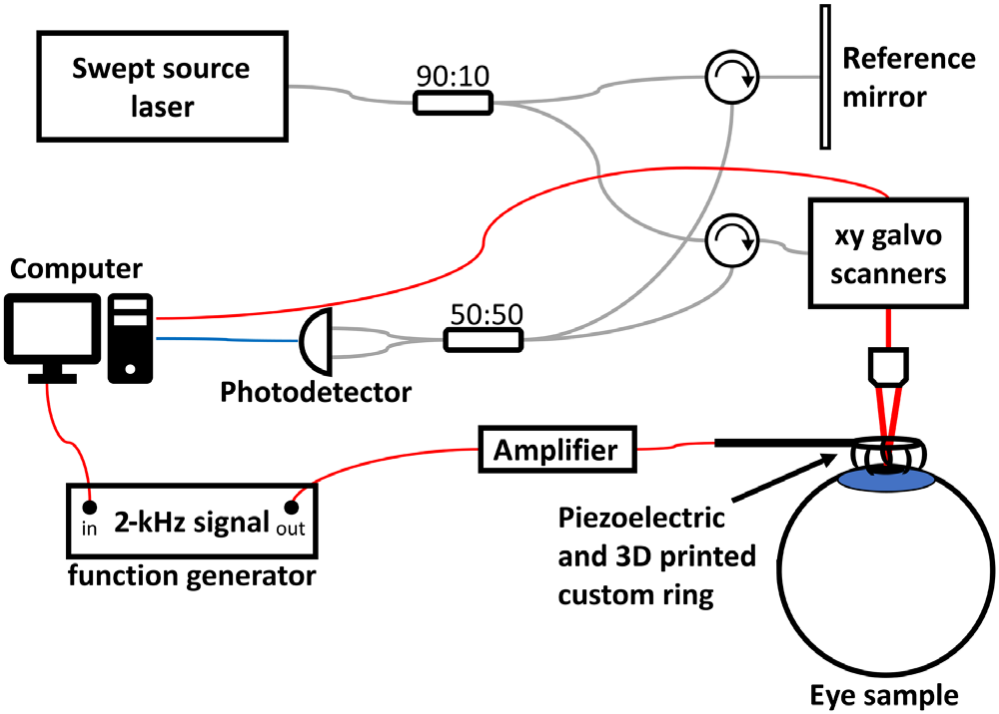
Experimental setup for Rev3D-OCE.

A custom phase-sensitive swept-source optical coherence tomography (SS-OCT) system is used with a synchronized mechanical excitation system. The SS-OCT system is implemented with a SS laser (HSL-2100-HW, Santec, Aichi, Japan) with a center wavelength of 1310 nm and a bandwidth of 140 nm. The lateral resolution is 20  μm, and the full-width half-maximum of the axial point spread function after dispersion compensation is 6  μm in air. The maximum sensitivity of the system was measured to be ∼110  dB. The imaging depth was 5 mm in air (with a sensitivity roll-off of −10  dB). The SS-OCT system and the synchronized mechanical excitation system are controlled together with LabVIEW (Version 14, National Instruments, Austin, Texas). The experimental setup is shown in Fig. 1.

**Fig. 2:**
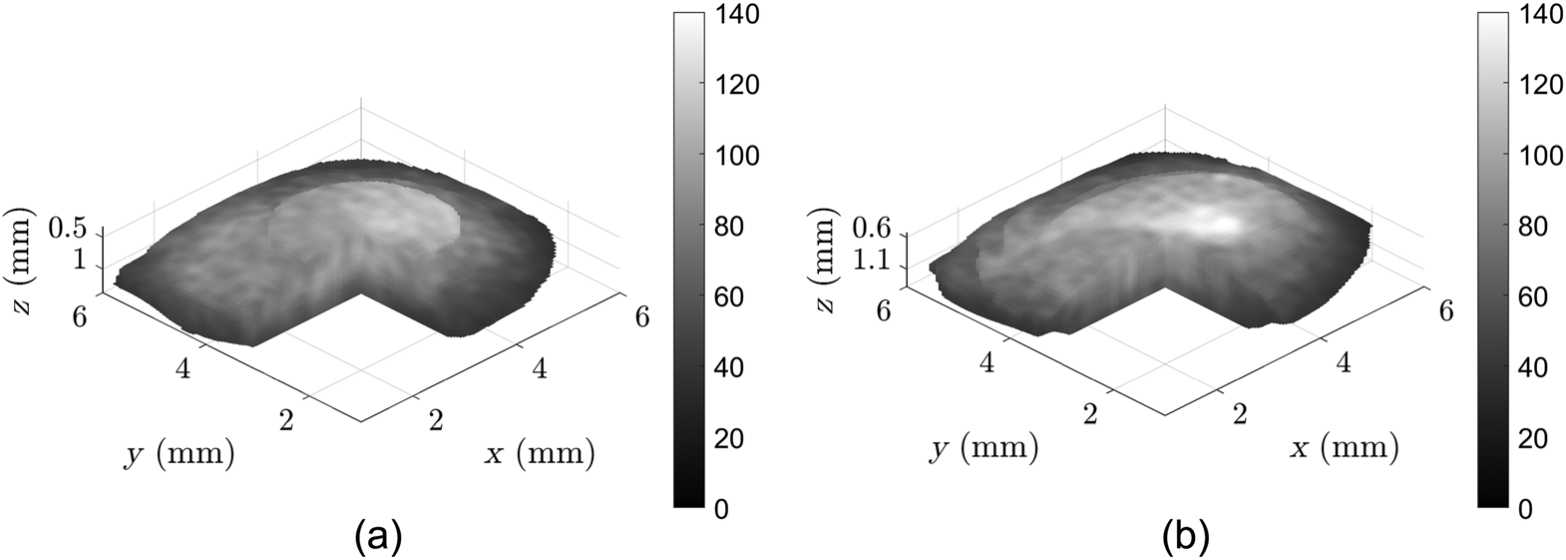
(a) Pre- and (b) post-CXL 3D B-mode scans of a sample pig cornea in a high-dose CXL experiment. Color bar in arbitrary grayscale units following log transformation of raw scans.

The mechanical excitation system includes a function generator (AFG320, Tektronix, Beaverton, Oregon) that provides the excitation output signal, an amplifier (PDu150, PiezoDrive, Callaghan, New South Wales, Australia), a piezoelectric actuator (BA4510, PiezoDrive, Callaghan, New South Wales, Australia), and a custom 3D-printed ring with eight concentric points of equidistant points of contact from the center with a 10-mm diameter aperture to allow for SS-OCT scans. The actuator is attached to the base of the custom ring. The custom ring is placed gently onto the cornea to induce mechanical shear waves using a custom mount. The mount is stable to ensure that the custom ring is aligned with the OCT system, i.e., the laser passes through the ring’s aperture. This actuator and ring configuration was pioneered by Zvietcovich et al.^18^ and is shown in Fig. 2 of their supplementary material. The function generator signal output was a continuous sinusoidal wave with a frequency of 2 kHz. The field of view (FOV) was defined to be a 6×6  mm area that contains the treatment zone in the center of the cornea.

### 2.3 Data Acquisition and Processing

The simultaneous M- and B-mode acquisition approach, as developed by Zvietcovich et al.,^18^^,^^19^ is used to acquire Rev3D-OCE data. One hundred (100) A-lines by 100 frames by 100 M-mode measurements were acquired, resulting in a four-dimensional matrix consisting of 3D space (or volume) and time dimensions. The estimated particle motions or phase differences were obtained using the algorithm developed by Loupas et al.^20^

The theory behind reverberant shear wave fields is briefly described in the Appendix. The two-dimensional (2D) spatial autocorrelations for each xy-plane are calculated with an approximate window size of 1×1  mm. The wavenumber k is then obtained through fitting. With the known input frequency of 2 kHz, the resulting shear wave speeds (SWSs) are estimated using the equations described in the Appendix. This process is repeated to construct the 3D elastograms (or SWS maps). The 3D regions of interest are obtained via active contouring for the upper surface and signal-to-noise ratio (SNR) thresholding for the lower surface. All data processing was completed using MATLAB 2020b (Mathworks, Natick, Massachusetts).

## 3 Results

### 3.1 Representative Samples

**Fig. 3:**
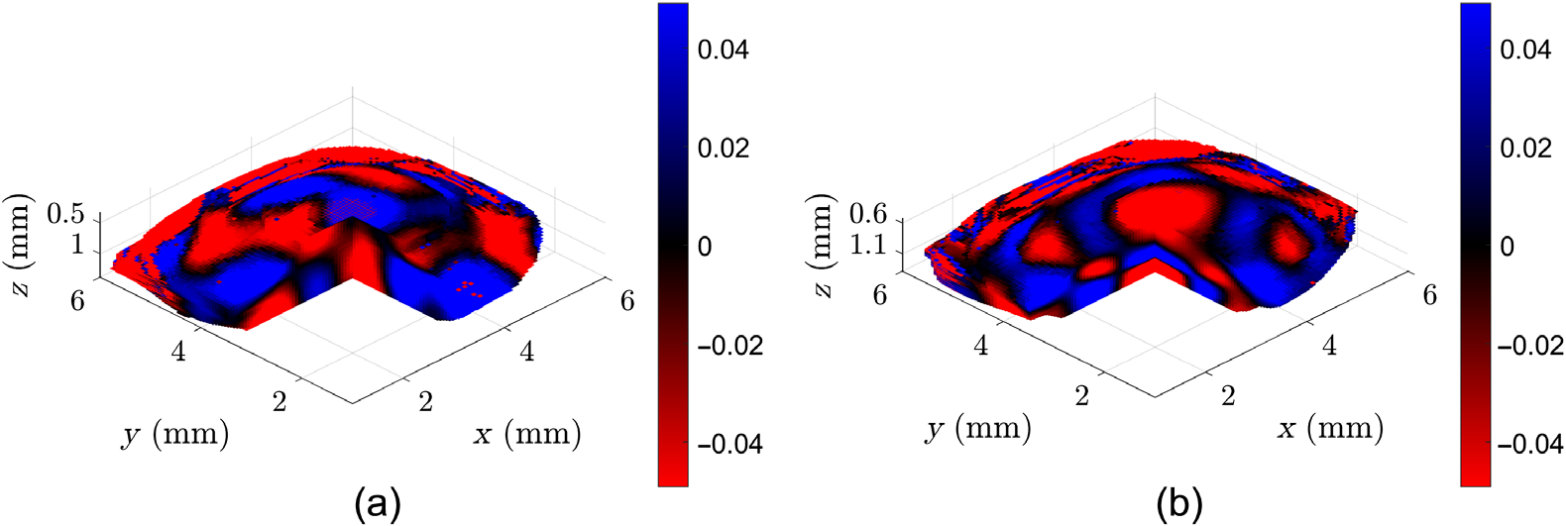
(a) Pre- and (b) post-CXL sample motion frames of a sample pig cornea in a high-dose CXL experiment. Color bar in arbitrary units of particle velocity.

**Fig. 4:**
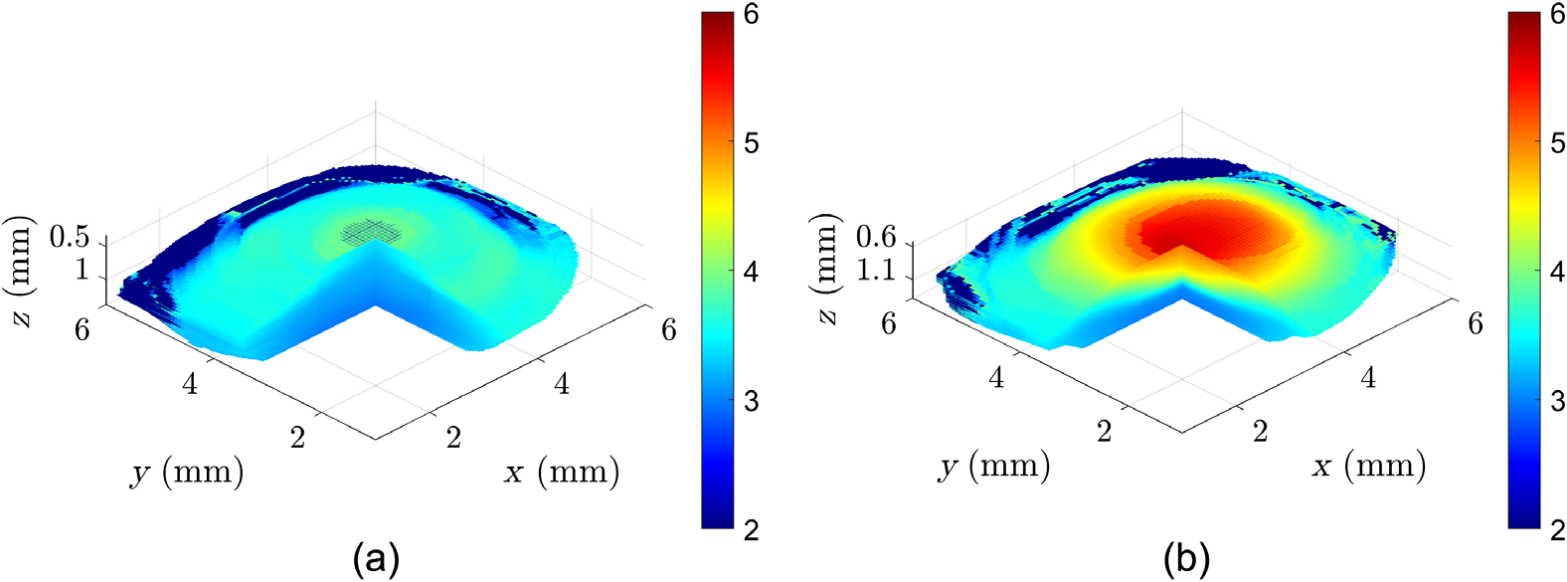
(a) Pre- and (b) post-CXL SWS maps of a sample pig cornea in a high-dose CXL experiment. Color bar units for the SWS are in m/s.

**Fig. 5:**
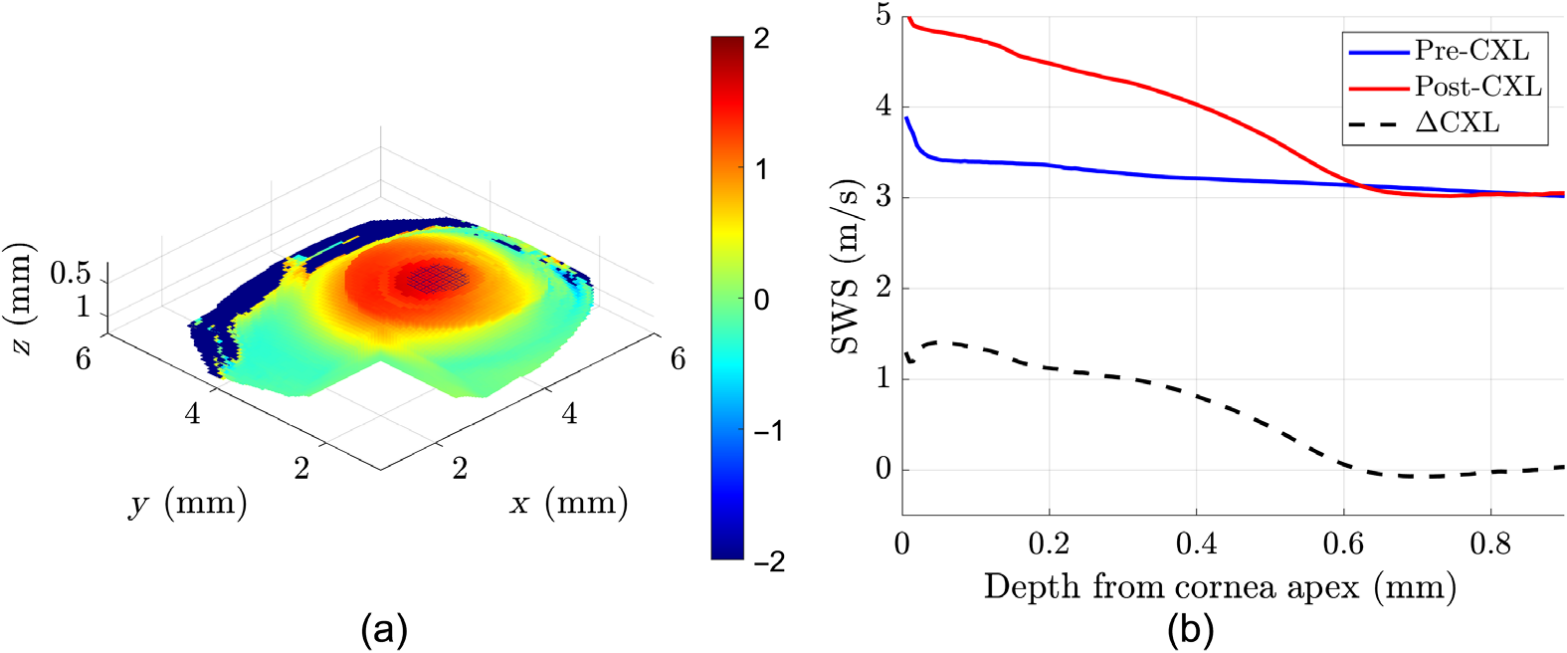
(a) 3D difference map created by centering and subtracting the two SWS maps in Fig. 4. Color bar units are in m/s. (b) Averaged SWS as a function of depth in the 3-mm treatment zone. The CXL procedure results in increased SWS. The difference between pre- and post-CXL profiles is denoted as ΔCXL.

In this section, a representative sample is shown to demonstrate the Rev3D-OCE technique in evaluating the CXL protocols. While the scanning area spans a 6×6  mm FOV, only voxels representing corneal tissue and with sufficient SNR are utilized to estimate the reverberant shear wave fields. Figure 2 shows the two 3D B-mode scans of a pig cornea pre- and post-CXL in a high-dose experiment, in which only valid voxels are shown with a pie-cut to demonstrate the interior. Sample frames of particle motion demonstrating the reverberant shear wave field phenomenon are shown in Fig. 3. Figure 4 shows the estimated SWS maps, also called elastograms. Figure 5 demonstrates the change in SWS due to the CXL procedure. No notable differences were found in the negative control experiment where the UV-A treatment was removed. This verifies that the elasticity changes seen are due to the CXL treatments, while the effect of drug application and the mitigated natural dehydration during experiments on stiffness changes showed to be negligible.

### 3.2 Summary of Experiments

**Fig. 6:**
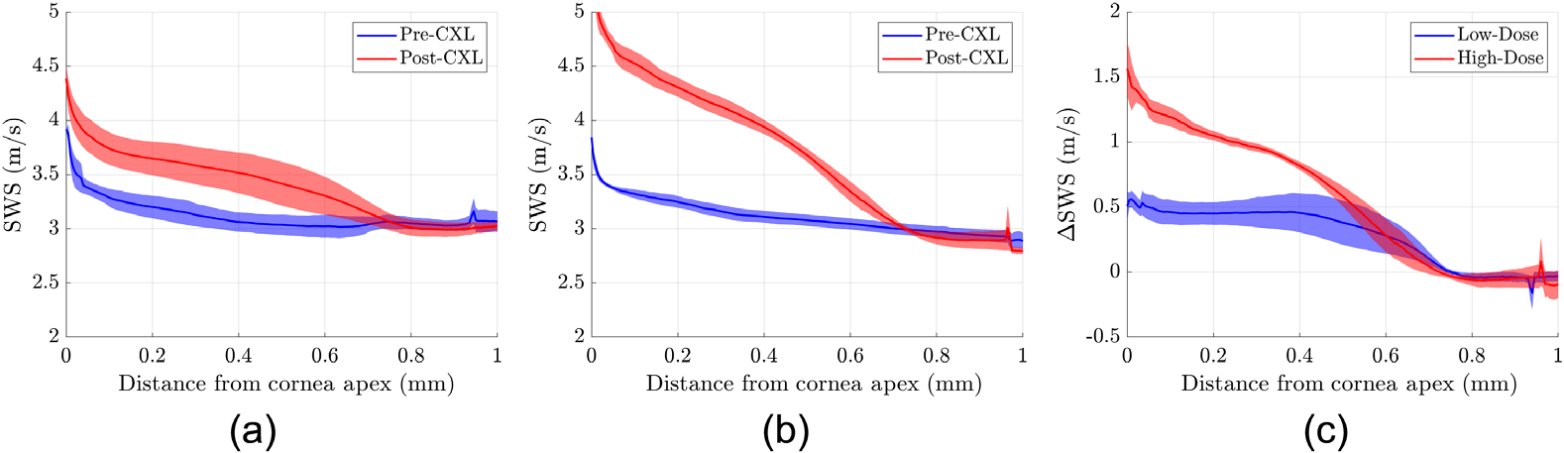
(a) SWS profiles for low-dose CXL experiments (n=3 eyes). (b) SWS profiles for high dose CXL experiments (n=3 eyes). (c) Increases in SWS profiles are compared between the low- and high-dose experiments. Average values are plotted with standard error of the mean in shaded red or blue.

Figure 6 summarizes the experiments performed at a frequency of 2 kHz. A total of six eyes (three pairs) were used for three low-dose and three high-dose CXL procedures.

## 4 Discussion and Conclusion

In Sec. 3.1, we show representative elastograms for a pig cornea before and after a high-dose CXL experiment. Figure 3 shows the increased apparent wavelength that is indicative of increased stiffness only in the center. As shown in Figs. 4 and 5, the SWSs are elevated in the 3-mm treatment zone. Figure 5(b) shows that the increased stiffness from the CXL procedure only affects the upper 0.6 mm of the cornea closest to the surface. Section 3.2 shows the summary of repeated experiments that demonstrate that CXL procedures result in increased stiffness of the upper 0.6 mm of corneal tissue. For Fig. 6, instead of using statistical tests, we show the standard errors to graphically demonstrate the lack of overlapping curves in the cornea’s initial 0.4 to 0.6 mm. While there are quantitative statistical tests to compare the similarity of shape and trends, no simple and direct test would indicate significant increases between curves without assuming the measurements along depth are independent. Notably, Figs. 5(b) and 6 show sharp nonlinear declines in SWS within the initial 0.1 mm of the cornea. The exact reason for this is unknown, but we will offer two potential explanations. The first is that if this effect is physical, it could be due to slight desiccation of the cornea at the surface. Even with chronic flushing of BSS, tissue dehydration is inevitable in *ex vivo* experiments, and it would be worse at the surface. The second explanation theorizes that the effect is computational since a high-intensity backscatter is associated with the corneal surface, which may skew the motion estimator and elastography analysis in that area. Overall, Rev3D-OCE is a promising high-resolution technique that can not only identify elasticity changes in CXL procedures, it can also identify depth-resolved changes in elasticity as well as the nonlinearity of changes. Further studies are needed to validate not only the elasticity values obtained, but also the depth of CXL observed.

The spatial resolution of elastography is dependent on a complex interaction of multiple factors, as discussed by Zvietcovich et al.^18^ Aside from furthering the resolution of the optical coherence tomography imaging system, we optimize the lateral elastography resolution for corneal CXL applications by varying the window size necessary to compute the 2D autocorrelation functions. Smaller window sizes offer increased spatial elastography resolution at the cost of accuracy. Elastography resolution along depth is fixed based on the depth resolution of the imaging system, but elasticity gradient characterization along depth relies on the accuracy of the measurements. For the CXL experiments, we used a window size that prioritized increased spatial resolution to better visualize CXL treatment boundaries. The subsequent decrease in accuracy is not as pertinent since relative comparisons are then performed. However, future studies will be needed to quantify, model, and optimize parameters of elastography resolution and accuracy.

When compared with previous elastography studies (using Rayleigh and Lamb wave models) of corneal CXL at similar UV irradiances and experimental conditions, the SWS measurements obtained pre- and post-CXL and SWS increases are in range with those reported in the literature.^8^^,^^13^^,^^14^^,^^16^ This is summarized in Table 1, where some measurements are converted or averaged (such as Young’s modulus). However, current studies report elasticity measurements for the entire cornea as a whole, and do not present corneal layer gradients. Thus, direct comparison of elasticity values as well as depth of CXL of these previous studies with the Rev3D-OCE method cannot be performed. Future studies with larger sample sizes are needed to evaluate and validate Rev3D-OCE with various CXL protocols in the clinical setting. Limitations of the technique currently include acquisition time and the fact that the technique requires physical contact with the cornea for shear wave induction. Finally, further refinements in the estimator used for Rev3D-OCE would allow for improved elastographic spatial resolution and accuracy.

**Table 1:** Summary of previous studies with similar experimental conditions for comparison.

Study model	Animal model	OCE method	Excitation frequency	Results [m/s] (converted/averaged)	Approximate SWS increase (m/s)
Lamb wave model^8^	Rabbit (*ex vivo*)	Air pulse	n/a	Untreated: 1.4	2.5
				CXL: 3.9	
Modified Rayleigh–Lamb (multifrequency)^13^	Porcine (*ex vivo*)	Air pulse	∼200−800 Hz	Untreated: 4.16	1.9
				CXL: 6.06	
Anisotropic model^14^	Porcine (*ex vivo*)	Air pulse	n/a	Untreated: 4.08	2.6
				CXL: 6.71	
Modified Rayleigh–Lamb (multifrequency)^16^	Rabbit (*ex vivo*)	Air pulse	∼200−800 Hz	Untreated: 4.93	1.1
				CXL: 6.01	

## 5 Appendix: Rev-3D OCE Theory

For a linear-elastic, homogeneous, and isotropic material, the quantity of interest is the elastic modulus E, which is related to the SWS $c_{s}$ in the sample as follows:

$$E=3\rho c_{s}^{2},$$ (1)

where ρ is the density of the material. Current elastography systems are prone to reflections from sample boundaries and inhomogeneities, which complicates SWS calculations. To obtain more accurate elastography measurements, we used the theory of reverberant shear wave fields, as derived by Zvietcovich et al.^18^ and Parker et al.^21^ This technique takes advantage of the inevitable reflections from boundaries and inhomogeneities by mathematically modeling them for the case of an isotropic distribution of shear waves propagating in all directions. This reverberant field produces a particle velocity vector field V(ε,t) at position ε and time t. A single axis scan from OCT is measured as $V_{z}(\varepsilon ,t)$. The spatial autocorrelation of $V_{z}(\varepsilon ,t)$, $B_{V_{z}V_{z}}$, along orthogonal directions is described as

$$B_{V_{z}V_{z}}=V_{\mathrm{avg}}^{2}[j_{0}(k\Delta \varepsilon_{x})-\frac{j_{1}(k\Delta \varepsilon_{x})}{k\Delta \varepsilon_{x}}],$$ (2)

where $V_{\mathrm{avg}}^{2}$ is the mean squared scalar value of particle velocity, $j_{0}$ and $j_{1}$ are the spherical Bessel functions of the first kind of zero- and first-order, respectively; k is the wavenumber and $\Delta \varepsilon_{x}$ represents displacement along an orthogonal x axis. By fitting autocorrelation profiles, and given a known excitation frequency ω, the SWS can be calculated as $c_{s}=\omega /k$. While the density, ρ, of the material can be assumed to be some quantity, it is conventional in the literature to directly report SWS values instead of estimated elastic moduli.

## Code, Data, and Materials Availability

All relevant code, data, and materials are available from the authors. Correspondence and requests should be addressed to the corresponding author.
